# Experimental Evolution Expands the Breadth of Adaptation to an Environmental Gradient Correlated With Genome Reduction

**DOI:** 10.3389/fmicb.2022.826894

**Published:** 2022-01-26

**Authors:** Masaomi Kurokawa, Issei Nishimura, Bei-Wen Ying

**Affiliations:** School of Life and Environmental Sciences, University of Tsukuba, Tsukuba, Japan

**Keywords:** experimental evolution, niche, local adaptation, global adaptation, growth fitness, culture medium, experimental ecology

## Abstract

Whether and how adaptive evolution adjusts the breadth of adaptation in coordination with the genome are essential issues for connecting evolution with ecology. To address these questions, experimental evolution in five *Escherichia coli* strains carrying either the wild-type genome or a reduced genome was performed in a defined minimal medium (C0). The ancestral and evolved populations were subsequently subjected to fitness and chemical niche analyses across an environmental gradient with 29 combinations of eight chemical components of the minimal medium. The results showed that adaptation was achieved not only specific to the evolutionary condition (C0), but also generally, to the environmental gradient; that is, the breadth of adaptation to the eight chemical niches was expanded. The magnitudes of the adaptive improvement and the breadth increase were both correlated with genome reduction and were highly significant in two out of eight niches (i.e., glucose and sulfate). The direct adaptation-induced correlated adaptation to the environmental gradient was determined by only a few genome mutations. An additive increase in fitness associated with the stepwise fixation of mutations was consistently observed in the reduced genomes. In summary, this preliminary survey demonstrated that evolution finely tuned the breadth of adaptation correlated with genome reduction.

## Introduction

Microorganisms living in nature show highly diverse habitats (i.e., ecological niches) as a consequence of local adaptation (Kawecki and Ebert, 2004) and are constrained by evolutionary costs (Bono et al., 2020). The ecological niche is believed to be associated with genomic information (Alneberg et al., 2020), which is considered to be a result of adaptive evolution (Batut et al., 2014). Numerous studies have reported adaptation to a certain niche related to genetic causes, such as linkages between genome streamlining and niche partitioning (Graham and Tully, 2021), gene loss and niche shifts (Chu et al., 2021), genome reduction and habitat transition (Salcher et al., 2019), metabolic costs (Ankrah et al., 2018), genome architecture and habitat (Getz et al., 2018) or niche-directed evolution (Andrei et al., 2019). These findings provide strong evidence linking adaptive evolution to ecological niches in terms of the spatial and environmental differentiation of species. As environmental changes are more often gradual under temporal and spatial restrictions, whether the breadth of adaptation to environmental gradients is shaped by evolution is an intriguing question.

Adaptation to a certain environment (i.e., niche) is often investigated by means of experimental evolution (Kawecki et al., 2012; Barrick and Lenski, 2013) to acquire direct evidence and preform a precise evaluation. In general, these studies have focused on a target component among the numerous components that comprise the environment, such as carbon sources (Satterwhite and Cooper, 2015) or antibiotics (Baym et al., 2016), as the factor triggering adaptive evolution. The environment, whether it is the culture medium used in the laboratory or the ecological niche in nature, is comprised of not only the target component but also a number of other nutrients and trace elements. Thus, adaptation must occur not only to the target component but also to all of the remaining components in the environment. However, the participation of components other than the target component in adaptive evolution has generally been neglected. A machine learning analysis of medium components showed that it was the trace elements (e.g., metal ions) rather than the major nutrients (e.g., glucose) that determined bacterial growth, which was sensitive to the concentration gradient (Ashino et al., 2019). Thus, whether and how adaptation through experimental evolution is associated with correlated adaptation to environmental gradients must be addressed.

In addition, genome size, as a quantitative index of genetic richness, has been intensively studied; nevertheless, its impact on adaptation remains unclear. Changes in genome size are commonly observed in nature (Kuo and Ochman, 2009; Batut et al., 2014; Maistrenko et al., 2020) and are known as one of the driving forces of adaptive evolution (e.g., horizontal gene transfer) (Keeling and Palmer, 2008; Daubin and Szöllősi, 2016). Genome size can be experimentally reduced (Posfai et al., 2006; Kato and Hashimoto, 2007; Mizoguchi et al., 2008) to determine the minimal genetic requirement of living organisms (Xavier et al., 2014; Rees-Garbutt et al., 2020). Such reduced genomes tend to show decreased fitness (Karcagi et al., 2016; Kurokawa et al., 2016) and increased mutagenesis (Nishimura et al., 2017), which can both be restored by experimental evolution (Nishimura et al., 2017). These studies have revealed that genome reduction not only has evolutionary consequences but also plays a role in adaptation. To date, the experimental evidence of the contribution of genome reduction to adaptation is largely insufficient.

**FIGURE 1:**
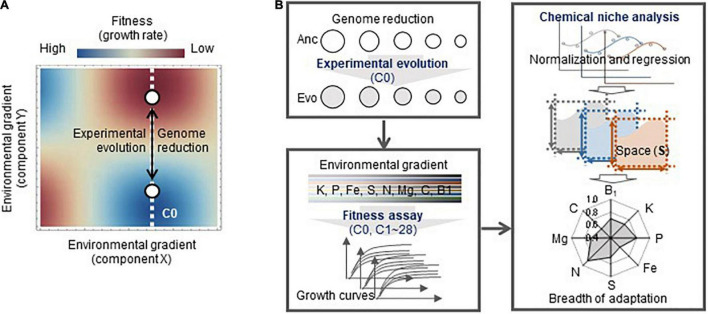
Conceptual illustration of the study. **(A)** Fitness landscapes of the reduced genomes in the environmental gradient. The evolution of the genomes (open circles), from Anc to Evo, leads to not only adaptation to the environment for evolution (C0, white broken line) but also correlated adaptation to the environmental gradient. The color gradation from red to blue indicates the fitness from high to low. **(B)** Overview of the experiments and analyses performed in the present study. The experimental and analytical studies are categorized in three boxes, which are the experimental evolution, high-throughput fitness assay, and chemical niche analysis. Five genomes (strains) used for the evolution are shown as circles. The eight elements investigated in the chemical niche analysis are indicated.

To investigate whether experimental evolution in a defined steady condition caused a change in the breadth of adaptation (Figure 1A), a pilot survey of the adaptation connecting genome reduction with environmental gradients was performed in the present study. Direct adaptation was achieved by experimental evolution, which was conducted with an assortment of laboratory *Escherichia coli* (*E. coli*) strains derived from the same parental wild-type genome with different genome sizes (Figure 1B). Whether and how direct adaptation contributes to correlated adaptation to an environmental gradient were quantitatively evaluated by fitness assays and chemical niche analysis. The impact of genome reduction on adaptation was analyzed in parallel, which provides the experimental demonstration and insight that connecting the two “unrelated” issues of correlated adaptation and genome reduction. The results filled the blank of knowledge on the breadth of adaptation and genome.

## Results

### Adaptation Correlated With Genome Reduction

**FIGURE 2:**
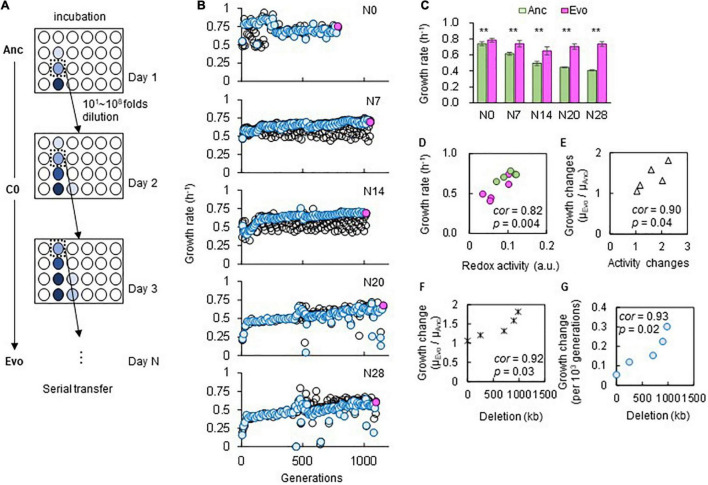
Experimental evolution for adaptation to C0. **(A)** Illustration of experimental evolution. The gradation from light to dark blue indicates the cell concentration from low to high. The *E. coli* cells were cultured in eight wells, and eight 10-fold serial dilutions were prepared. Only one of the eight wells (dilutions) showing growth in the early exponential phase was used for serial transfer, as indicated by dotted boxes. **(B)** Temporal changes in growth rates during the experimental evolution. The five genomes are indicated. The blue circles represent the lineages adopted for the following serial transfer, corresponding to the wells indicated by dotted boxes in **(A)**. The black circles stand for the remaining serial dilutions (i.e., the other seven wells) that were not adopted for serial transfer. The endpoint populations of the evolutionary lineages (blue circles) used for the following analyses are indicated in pink. **(C)** Growth rates in the medium for evolution. Green and pink bars represent the growth rates of Ancs and Evos in C0, respectively. Standard errors of biological replications (*N* > 6) are indicated. Asterisks indicate the statistical significance of the two-tailed Student’s *t*-test (*p* < 0.01). **(D,E)** Correlation between the growth rate and cellular redox activity. Green and pink circles represent Ancs and Evos, respectively. The Spearman rank correlation coefficients and statistical significance are indicated. **(F)** Correlation between the changes in growth rate and genome reduction. The lengths of the genomic deletions are plotted against the ratio of the growth rates of Ancs and Evos. The Spearman rank correlation coefficients and statistical significance are indicated. **(G)** Correlation between the changes in growth rate per generation and genome reduction. The Spearman rank correlation coefficients and statistical significance are indicated.

The adaptation of the genomes of different sizes was consistently achieved through experimental evolution under stable conditions. Five laboratory *E. coli* strains carrying either a wild-type (N0) or reduced (N7, 14, 20, or 28) genome (Supplementary Table 1) were subjected to experimental evolution in a chemically defined medium (C0). The evolution experiment was performed by serial transfer at a series of different dilution rates to maintain bacterial growth within the exponential phase (Figure 2A and Supplementary Figure 1). The daily records showed that a gradual increase in the growth rate consistently occurred in the reduced genomes (Figure 2B), and the population that proceeded (blue) along the dilution series (black) presented a somewhat rapid fitness increase. The evolutionary trajectories of the reduced genomes were somehow similar, in comparison to that of the wild-type genome. It was unclear whether the evolutionary path was attributed to the deletion of genomic sequences or the relatively low fitness of the ancestor population. The improved fitness of the endpoint population indicated that adaptation to C0 was achieved, which was highly significant in the lineages of reduced genomes. It should be noted that the fluctuation in the growth rate recorded in N20 and N28 was largely due to a pause of serial transfer, which was restarted from the glycerol stock of the bacterial population stored the day before.

Fitness and activity assays showed that adaptation was correlated with genome reduction. The comparison of the ancestors (Ancs) and the evolved populations (Evos) after approximately 1,000 generations (Figure 2B, pink) showed that the growth rates of Evos were all significantly higher than those of Ancs (Figure 2C). Positive correlations were observed between the growth rates and redox activities (Figure 2D) as well as between the changes thereof (Figure 2E), which verified that direct adaptation was achieved at both the growth and metabolic levels. Genome reduction-correlated changes in the growth rate were identified (Figure 2F). The evolutionary rate of the changes in the growth rate was correlated with genome reduction (Figure 2G), which was consistent with the correlation between genome reduction and the spontaneous mutation rate (Nishimura et al., 2017), a global parameter representing evolvability.

### Direct Adaptation-Mediated Correlated Adaptation to the Environmental Gradient

**FIGURE 3:**
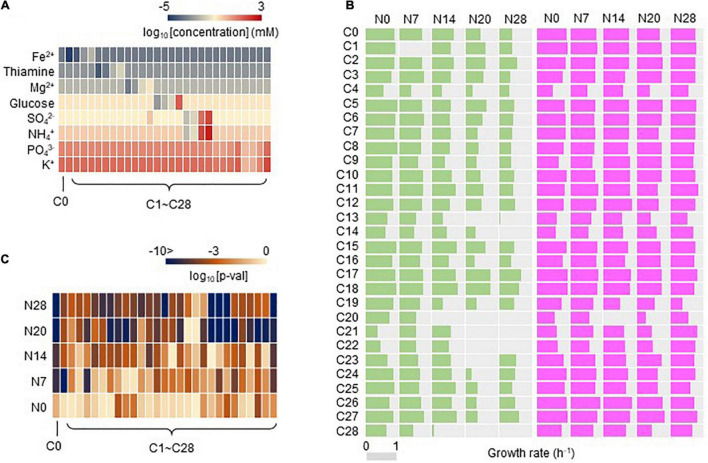
Growth rates evaluated in 29 medium combinations. **(A)** Heatmap of the concentration gradient. The eight chemical components of the media are indicated. The gradation from red to blue indicates the concentration, which is shown on the logarithmic scale, from high to low. C0 is the environment for evolution. **(B)** Growth rates of Ancs and Evos in the environmental gradient. The growth rates evaluated in the 29 medium combinations are shown. Pink and green represent Evos and Ancs, respectively. The gray bar represents the scale of the exact growth rate (h^– 1^). **(C)** Heatmap of statistical significance of the changes in growth rate of Evos and Ancs. The gradation from light orange to dark blue indicates the *p*-value, which is shown on the logarithmic scale, from low to high.

Whether the adaptation to C0 caused adaptation or maladaptation across the environmental gradient was further evaluated. A total of 29 medium combinations (C0, C1∼28) were prepared with seven pure chemical substances that were included in C0 (Supplementary Table 2). These combinations comprised eight constituents (e.g., ions), whose concentrations varied broadly on a logarithmic scale (Figure 3A). Adaptiveness, represented by the growth rates in the exponential phase, was evaluated according to a total of 2,220 growth curves (Supplementary Table 3). Overall, a global increase in the growth rate across the environmental gradient of the 29 combinations was detected in the Evos with the reduced genomes (Figure 3B, pink) in comparison to the common decrease in the growth rate of Ancs (Figure 3B, green). The increased growth rates of the Evos with the reduced genomes were highly significant in most tested combinations (Figure 3C and Supplementary Table 4). This result indicated that adaptation was achieved not only specific to C0, but also globally, to the environmental gradient.

**FIGURE 4:**
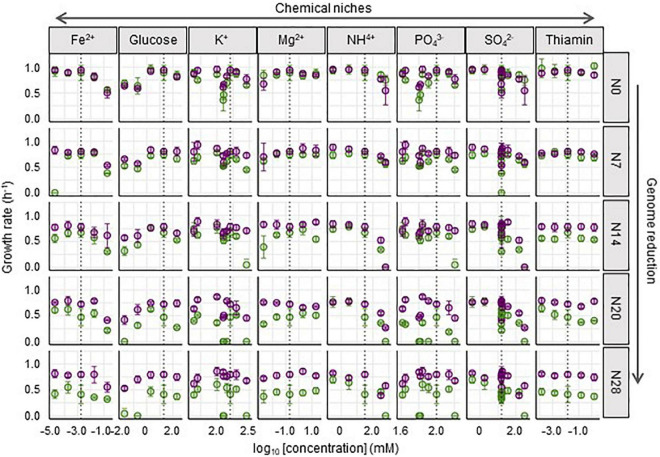
Fitness across the concentration gradient of individual chemical niches. The mean growth rates in the 29 medium combinations are shown. The concentrations of chemical niches are shown on a logarithmic scale. The wild-type and reduced genomes are indicated as N0 and N7∼N28, respectively. Purple and green represent Evos and Ancs, respectively. Standard errors of biological replications (*N* > 6) are indicated.

Whether and how such correlated adaptation was determined by any of the eight constituents was analyzed. The growth rates of Ancs and Evos in each constituent were plotted in response to the concentration gradient (Figure 4). A larger reduction in the genome was likely to be associated with larger changes in the growth rate between Anc and Evo, regardless of the variation in the constituents. In contrast, the patterns of the changes in the growth rate were dependent on the constituents. Different patterns (dynamics) among the eight constituents were consistently observed in all genomes, suggesting that the breadth of adaptation was chemically dependent, (i.e., niche dependent).

### Niche-Specific and Genome Reduction-Correlated Expansion of the Breadth of Adaptation

**FIGURE 5:**
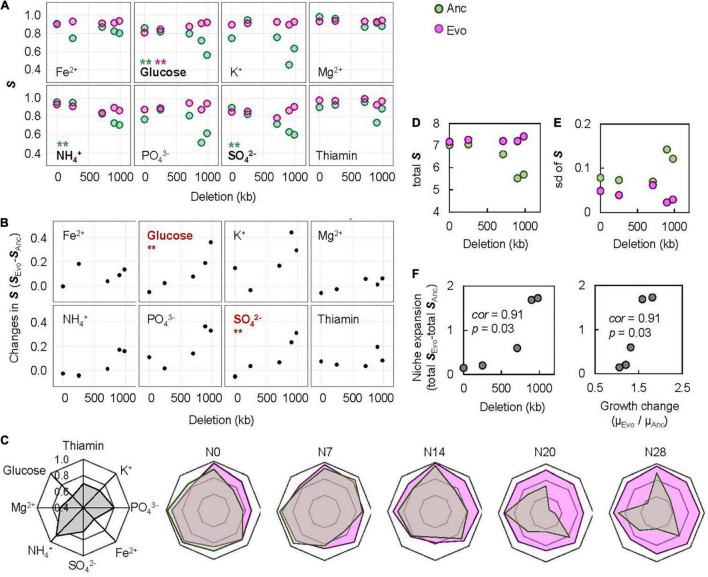
Evaluation of the breadth of adaptation. **(A)** Relationships between the ***S*** and genome reduction. The chemical niches and the statistical significance of the Spearman rank correlation are indicated. Boldfaces associated with asterisks represent statistical significance (**p < 0.01). Pink and green represent Evo and Anc, respectively. **(B)** Relationships between the changes in ***S*** and genome reduction. The chemical niches and the statistical significance of the Spearman rank correlation are indicated. Boldfaces in red associated with asterisks represent statistical significance (**p < 0.01). **(C)** Radar chart of the eight niche spaces. The eight chemical niches and the scale of ***S*** are illustrated in the monotone radar chart. The five genomes are shown separately. Transparent pink and green represent Evos and Ancs, respectively. **(D)** Sum of the eight ***S***. **(E)** Standard deviation of the eight ***S***. **(F)** Correlation of the changes in total ***S*** to genome reduction and direct adaptation. The Spearman rank correlation coefficients and statistical significance are indicated.

To evaluate the breadth of adaptation, representing the equitability of fitness along the environmental gradient (Lynch and Gabriel, 1987), the space (***S***) was newly defined as the shadowed space under the fitting curve of cubic polynomial regression to the normalized dynamic of change in growth rate according to chemical concentration, in which the maxima of both the concentration gradient and the growth rate were rescaled to one unit (Supplementary Figure 2). Normalization and regression suggested a global parameter of ***S*** available for quantitative comparison among the varied chemical niches and genomes, where a larger ***S*** indicated a wider breadth of adaptation. A total of 80 ***S*** values (Supplementary Figure 3) and 40 changes in ***S*** (Supplementary Figure 4) were calculated accordingly. A correlation of genome reduction with the ***S*** of Ancs in the chemical niches of glucose, SO_4_^2–^ and NH_4_^+^ was observed (Figure 5A, green). Additionally, a correlation of genome reduction with the evolutionary changes in ***S*** in the chemical niches of glucose and SO_4_^2–^ (Figure 5B) was identified. The correlation with genome reduction could also account for direct adaptation (Supplementary Figure 5), as it was accompanied by genome reduction (Figure 2). The chemical niches related to carbon and sulfate were likely to be highly essential and sensitive for adaptation.

The overall improvement of the breadth of adaptation to the eight chemical niches was evaluated by the total ***S***, which was determined as the sum of the eight ***S*** values. It was narrowed in response to genome reduction but significantly enlarged due to experimental evolution (Figure 5C). The total ***S*** of Evos entirely encompassed that of Ancs among the bacteria with reduced genomes with larger deletions (N14, N20, and N28) but partially overlapped in the bacteria with a wild-type genome (N0) or a reduced genome with a relatively small deletion (N7). Taking N0 as an example, the increase in the ***S*** of thiamine, K^+^, PO_4_^3–^, and Fe^2+^ and the decrease in the ***S*** of glucose, Mg^2+^, NH_4_^+^, and SO_4_^2–^ implied that the improvement in the breadth of adaptation was chemical niche dependent and directional. In contrast, the omnidirectional expansion of total ***S*** occurred in the reduced genomes of N14, N20 and N28, which indicated general adaptation to the eight chemical niches. The adaptation to C0 restored the total ***S*** of all Evos to a level roughly equivalent to that of the wild-type genome (Figure 5D), indicating homeostasis in the adaptation to environmental gradients. The variation in ***S*** among the eight niches mostly declined in Evos (Figure 5E), indicating a balance in the breadth of adaptation to varied chemical niches. In addition, the changes in total ***S*** were positively correlated with direct adaptation and genome reduction (Figure 5F). These results indicated that the direct adaptation of *E. coli* expanded the breadth of adaptation to the chemical niches constituting the environment for homeostatic and balanced adaptation to the habit.

### Improved Adaptiveness Attributed to Stepwise Mutation Accumulation

**FIGURE 6:**
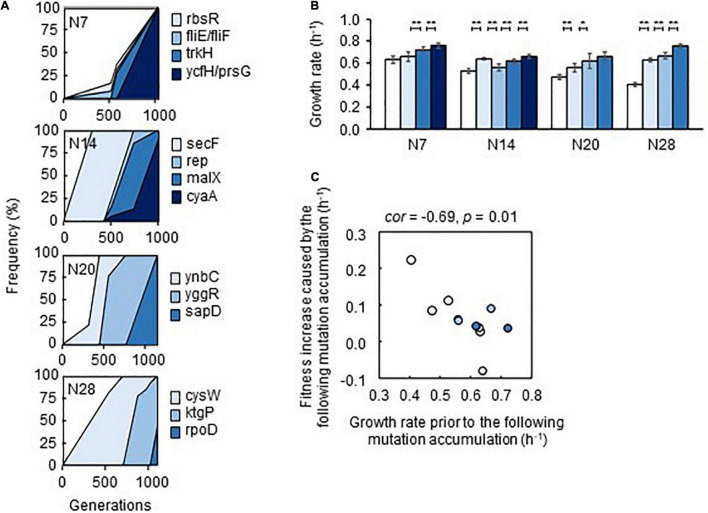
Fitness increase attributed to the mutations. **(A)** Stepwise accumulation of genome mutations. Mutation during the evolution. The temporal changes in the mutations fixed during the evolution are shown. The four reduced genomes and the gene names of mutants are indicated. **(B)** Additive increase in the growth rates of the mutants in the medium for evolution. Gradation from white to dark blue indicates the mutants with respect to those shown in **(A)**. Standard errors of biological replications (*n* > 6) are indicated. Asterisks indicate the statistical significance of the two-tailed Student’s *t*-test (*p < 0.05; **p < 0.01). **(C)** Correlation between mutation accumulation and changes in growth rates. A total of 12 mutants of a single accumulated mutation are shown. The Spearman rank correlation coefficients and statistical significance are indicated.

Genome mutation analysis (Supplementary Table 5) detected an approximately equivalent number of gene mutations in Evos (Supplementary Table 6). It should be noted that the deletion of transposons was ignored, and the mutations fixed in Evos were identified in the reduced genomes but not in the wild-type genome. The temporal changes in the allele frequency of mutants consistently showed that the mutations accumulated serially and were fixed in a stepwise manner (Figure 6A). Intriguingly, only a few mutations compensated for the genome reduction, independent of the degree of genome reduction. Abundant genetic information could be substituted with the modification of certain gene functions for equivalent adaptiveness, providing intriguing insight into genetic requirements. The findings were consistent with a previous report that a few mutations could cause the metabolic rewiring of a reduced genome (Choe et al., 2019). The majority of mutated genes were related to transporters and regulators, which indicated that resource diffusion for utilization and global gene regulation contributed to adaptation to the environmental gradient.

How the stepwise accumulation of the mutations contributed to adaptation was further investigated. The mutants carrying mutations in the order of their evolutionary accumulation were successfully acquired by single-colony isolation. A gradual increase in the growth rate of the mutants in the order of mutation accumulation was generally observed (Figure 6B), except for a transient decrease caused by the second mutation that occurred in N14. This demonstrated that the mutations were beneficial and contributed to adaptation in an additive manner. Notably, mutants with the second mutation (*rbsR* and *fliE/fliF*) in N7 failed to be acquired, indicating the co-fixation of the second mutation (*fliE/fliF*) and third mutation (*trkH*) during evolution. Intriguingly, a negative correlation between the growth rate and degree of fitness increase due to mutation was observed; that is, lower growth rates prior to mutation fixation led to greater changes in the growth rate after the mutation was fixed (Figure 6C). The first mutations were more likely to improve growth fitness than the mutations that were fixed later, although the statistical significance was weak (Supplementary Figure 6) because there were too few mutations. This finding agreed well with the rule of declining adaptability (Couce and Tenaillon, 2015) and the predictivity of the mutation-mediated fitness landscape (de Visser and Krug, 2014; Fragata et al., 2019).

### Proposed Mechanism of Direct Adaptation-Induced Niche Expansion

**FIGURE 7:**
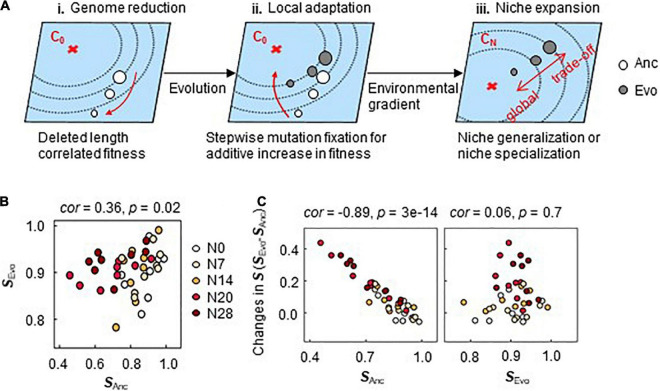
Mechanism for pre-adaptation. **(A)** Illustration of fitness landscape analysis. The fitness landscapes in environment C_0_ for the evolution and the alternative environments C_*N*_ are shown as the planes in blue. The red cross and the contour lines indicate the fitness peak (maximum) and the fitness gradient, respectively. The open and closed circles indicate the ancestor and evolved genomes, respectively. The size of the circles indicates the genome size. **(B)** Correlated **S** of Ancs and Evos. The color variation from white to dark red represents the five different genomes of N0∼N28. The niche spaces of Ancs and Evos are named ***S*_*Anc*_** and ***S*_*Evo*_**, respectively. **(C)** Relationships of ***S*_*Anc*_** and ***S*_*Evo*_** to the changes in ***S***. The changes in ***S*** caused by the evolution are plotted against ***S*_*Anc*_** and ***S*_*Evo*_** in the left and right panels, respectively. The Spearman rank correlation coefficients and statistical significance are indicated.

Fitness landscape analysis (Tenaillon, 2014; Martin and Lenormand, 2015), which is applied to explain mutation occurrence (Nahum et al., 2015; Bajic et al., 2018) with a change in distance to the fitness peak (Barrick et al., 2010; Schick et al., 2015) as an evolutionary constraint (Szamecz et al., 2014; Nahum et al., 2015), was employed to explain the present findings (Figure 7A). According to previous reports (Konstantinidis and Tiedje, 2004; Kurokawa et al., 2016), larger deletions in the genome lead to a greater distance from the fitness peak (**i**). Since direct adaptation was achieved in correlation with genome reduction (Figure 2), a greater distance from the fitness peak meant that a larger change was required to achieve equivalent fitness (**ii**). The fitness increase was additive owing to the stepwise fixation of gene mutations (Figure 6). Since direct adaptation expanded the breadth of adaptation to the environmental gradient (Figures 3–5), the location in the initial fitness landscape (e.g., distance from peak of C_0_) likely determined the adaptiveness to the alternative environmental gradient (e.g., C_*N*_) (**iii**). That is, there was a higher probability of an adaptive trade-off in C_*N*_ when Anc was located closer to the adaptive peak of C_0_ and a higher probability of correlated adaptation when Anc was located farther from the adaptive peak of C_0_. This mechanism was consistent with the pleiotropic costs of carbon utilization found in the experimental population (Jasmin and Zeyl, 2013).

Locational bias in the initial fitness landscape (Figure 7A) might cause so-called preadaptation (Cullum et al., 2001) to alternative environments. A weak but statistically significant correlation of ***S*** was detected between Ancs and Evos across the genomes and chemical niches (Figure 7B). The changes in ***S*** were highly significantly correlated with the ***S*** of Ancs but not with those of Evos (Figure 7C), even when evolutionary generation was taken into account (Supplementary Figure 7). Preadaptation might have participated in direct adaptation-induced niche expansion. Further evidence and investigation will be required to draw a solid conclusion.

## Discussion

Direct adaptation mediated by experimental evolution (Figure 2) was found to trigger correlated adaptation to the environmental gradient, consistently in five *E. coli* strains carrying the reduced genomes of size variation (Figures 3–5). This conclusion was drawn from a quantitative evaluation with the newly defined parameter of total ***S***, according to the fundamental niche concept proposed by Hutchinson (1957), which describes a niche without species’ interaction and is considered a high-dimensional hypervolume formed by environmental variables. In the present study, the variables were the eight constituents of the culture medium used for evolution (i.e., the chemical niches). The overall fitness equality across the environmental gradient seemed to be homeostatic in a defined habitat, as the total ***S*** increased while its variation decreased to comparable levels in five genomes of different reduced lengths (Figures 5C–E). A habitat composed of multiple chemical niches might decide the maximal accessibility for evolution. Notably, the homeostasis of niche space was not biased by normalization. As normalization to each single unit was performed individually, the maxima of the overall ***S*** could be differentiated among the respective genomes.

It was intriguing to find that correlated adaptation to the environmental gradient resulted from evolution under stable conditions. Since experimental evolution was conducted under stable conditions and bacterial growth was maintained in the exponential phase, neither nutritional starvation nor large environmental fluctuation was assumed to occur. As adaptation to one environment often results in maladaptation to alternative environments (Goddard and Bradford, 2003; Rodriguez-Verdugo et al., 2014; Satterwhite and Cooper, 2015), ecological niche speciation is often explained by adaptive trade-offs (Cooper and Lenski, 2000; Goddard and Bradford, 2003; Sexton et al., 2017; Chavhan et al., 2020). In evolution, environmental homogeneity is considered one of the factors determining trade-offs (Bono et al., 2017) and environmental fluctuation is thought to be crucial for adaptation to a wide range of environments (Wang and Dai, 2019). The reduced genomes evolved in a jack-of-all-trades-and-master-of-all manner, which has been proposed as one of three mechanisms of specialization that is widespread in nature (Remold, 2012); in contrast, the wild-type genome adopted a trade-off mechanism (Figures 5A–C), which is generally explained by constraints in phenotypic space (Shoval et al., 2012; Fraebel et al., 2017). In nature, the trade-off strategy might be more common and feasible for costless adaptation and niche expansion during eco-evolution (Ferenci, 2016; Farahpour et al., 2018; Bono et al., 2020).

The correlation between ***S*** and genome reduction was significant for glucose, NH_4_^+^ and SO_4_^2–^, whereas direct adaptation abolished this correlation for NH_4_^+^ and SO_4_^2–^ and changed the correlation from negative to positive for glucose (Figures 5A,B). Direct adaptation might directly compensate for the deficiency in using these resources, which seems reasonable because carbon, nitrogen and sulfur are the essential major elements required by living organisms on Earth (Wackett et al., 2004). Such niche specificity might reflect the evolutionary direction of generalists or specialists (Kassen, 2002; Bono et al., 2020). A large omnidirectional expansion of total ***S*** was found in the largely reduced genomes, in comparison to the small directional expansion of total ***S*** in the complete and slightly reduced genomes (Figure 5C). This revealed that organisms that experience large genome reduction evolved for generally and less genome reduction evolved for specially, which was an intriguing strategy of genome evolution for niche expansion.

Additionally, genome reduction-dependent features were generally observed. If any functions or mechanisms of the deleted genes were specifically responsible for direct and/or correlated adaptation, the size of the genome reduction would never be correlated with the fitness increase (Figure 2) or niche expansion (Figure 5). Genome reduction determines correlated adaptation to environmental gradients to some extent. A limitation of the study was lack of replication of experimental evolution, that is, only a single lineage of experimental evolution was applied in the five genomes. Nevertheless, the correlations of genome reduction to adaptation were highly reliable, because a non-correlated relationship will more frequently be acquired by chance. As all the correlations observed in the present study were statistically significant, additional evolutionary lineages would neither change the conclusion nor mask the correlations. Repeated experimental evolution is required to verify the generality of the correlations. Mutations appeared in the adaptive evolution might be fixed occasionally, as they were largely differentiated in gene functions and/or mechanisms (Supplementary Table 6). Repeated evolution experiments might result in different mutations in the same genome. This was the reason why the contribution of the mutations to fitness was analyzed from the viewpoint of correlation instead of gene function, which was assumed to be highly differentiated among multiple evolutionary lineages. The present study revealed a quantitative relationship among the genome reduction, adaptation and niche expansion as an experimental demonstration of the linkage between evolution and ecology. Further evaluation of the growth fitness in largely different environments will be highly valuable to clarify the evolutionary trade-offs and/or generality due to genome reduction.

## Materials and Methods

### *Escherichia coli* Strains

A total of five *E. coli* strains with either the wild-type or the reduced genome were used, which were selected from the KHK collection (Mizoguchi et al., 2008), an *E. coli* collection of reduced genomes (from National BioResource Project, National Institute of Genetics, Shizuoka, Japan). The wild-type and four reduced genomes were derived from *E. coli* W3110 and were assigned as N0 and N7, 14, 20, 28, respectively (Supplementary Table 1), according to previous studies (Kurokawa et al., 2016; Nishimura et al., 2017). Note that genome reductions are additively cumulative. Therefore, the higher numbered strains include all genomic reduction in the lower numbered strains.

### Media Combinations

The minimal medium M63, equivalent to C0, was used for the experimental evolution for direct adaptation. Its chemical composition was described in detail previously (Kurokawa et al., 2016; Kurokawa and Ying, 2017). The concentration gradient of the components of the M63 medium was prepared just before the fitness assay by mixing the stock solutions of individual chemical compounds, which resulted in 28 alternative medium combinations (C1∼28). The stock solutions, that is, 1 M glucose, 0.615 M K_2_HPO_4_, 0.382 M KH_2_PO_4_, 0.203 M MgSO_4_, 0.0152 M thiamin/HCl, 0.0018 M FeSO_4_, and 0.766 M (NH_4_)_2_SO_4_, were sterilized using a sterile syringe filter with a 0.22-μm pore size hydrophilic PVDF membrane (Merck, United States). The concentrations of most chemical compounds were altered one-by-one on a logarithmic scale to achieve a wide range of environmental gradients, as described previously (Ashino et al., 2019), which led to a total of 28 combinations. Both the medium used in the evolution (C0) and the alternative medium combinations (C1∼28) were used for the fitness assay. The resultant concentrations of individual components in the ionic form are summarized in Supplementary Table 2.

### Experimental Evolution

The experimental evolution of the five *E. coli* strains was performed within the early exponential phase by serial transfer, which was performed with 24-well microplates specific for microbe culture (IWAKI, Japan) as previously described (Nishimura et al., 2017). The *E. coli* cells were cultured in eight wells, and eight 10-fold serial dilutions, i.e., 10^1^∼10^8^, were prepared with fresh medium. The microplates were incubated overnight in a microplate bioshaker (Deep Well Maximizer, Taitec, Japan) at 37°C, with rotation at 500 rpm. Serial transfer was performed at 12- or 24-h intervals, according to the growth rate. Only one of the eight wells (dilutions) showing growth in the early exponential phase (OD_600_ = 0.01–0.1) was selected and diluted into eight wells of a new microplate using eight dilution ratios. The cell culture selected daily for the following serial transfer was mixed with glycerol (15% v/v) and stored at –80°C for future analyses. Serial transfer was repeatedly performed for approximately 50 days. The evolutionary generation was calculated according to the following equation (Eq. 1).


(1) $$g\,e\,n=l\,o\,g_{2}\,\left(C_{i}/C_{j}\right)$$

Here, *C*_*i*_ and *C*_*j*_ represent the OD_600_ of the cell culture that was used for serial transfer and the theoretical OD_600_ of the cell culture at the start of incubation. *C*_*j*_ was calculated by dividing the OD_600_ that was used in the last transfer by the dilution rate. To benefit experimental replication, the cell cultures stored for the following assays were dispensed into 20 microtubes in small aliquots (100 μL per tube), which were used once, and the remainder was discarded, as previously described (Kurokawa and Ying, 2017). Growth rate was estimated at every serial transfer according to the following equation (Eq. 2).


(2) $$\mu =L\,N\,\left(C_{i}/C_{j}\right)/\left(t_{i}-t_{j}\right)$$

Here, *C*_*i*_ and *C*_*j*_ are as described above. *t*_*i*_ is the time of serial transfer operation, and *t*_*j*_ is the time of the serial transfer operation immediately before *t*_*i*_.

### Fitness Assay

The fitness was determined as the maximal growth rate, as previously reported (Kurokawa et al., 2016). In brief, the cell culture stocks were diluted 1,000-fold in fresh media (C0, C1∼28) and were subsequently loaded into a 96-well microplate (Costar, United States) in six wells at varied locations. The 96-well microplate was incubated in a plate reader (Epoch2, BioTek) with a rotation rate of 567 rpm at 37°C. The temporal growth of the *E. coli* cells was detected by measuring the absorbance at 600 nm, and readings were obtained at 30-min intervals for 48 h. The maximal growth rate was calculated according to the following equation (Eq. 3).


(3) $$\mu =L\,N\,\left(C_{i+1}/C_{i}\right)/\left(t_{i+1}-t_{i}\right)$$

Here, *C*_*i*_ and *C_*i*+1_* represent the two reads of OD_600_ values at two consecutive time points of *t*_*i*_ and *t_*i*+1_*. The growth fitness was the average of the five continuous growth rates that exhibited the largest mean and the smallest standard deviation during the temporal changes in growth rate, as previously reported (Kurokawa et al., 2016). A total of 2,220 growth curves were acquired, and the corresponding growth rates were calculated for the analysis (Supplementary Table 3). Statistical significance of the changes in growth rate mediated by experimental evolution was evaluated by t-student test and the results are summarized in Supplementary Table 4.

### Redox Activity Assay

A cell culture in the exponential phase of growth (OD_600_ = 0.01 ∼ 0.3) was used for the assay. The cell culture was diluted with fresh medium at 12 dilution ratios from 1.75^0^ to 1.75^11^ in a final volume of 2 mL. Every 100 μL of the diluted cell culture was transferred to multiple wells in a 96-well microplate (Costar, United States), in which 20 μL of CellTiter 96^®^ Aqueous One Solution Reagent (Promega) was added. The reduction of the tetrazolium compound in the reagents was measured with a microplate reader (Epoch2, BioTek, United States) by determining the OD_490_ every 2 min for 30 min. The rate of reduction was calculated by linear regression of the temporal changes in OD_490_, i.e., the slope of the increase in OD_490_ over time (min). The redox activity was determined by dividing the rate of reduction by the OD_600_ of the cell culture. The mean of the multiple measurements (*N* = 5) was used for the analysis.

### Evaluation of the Breadth of Adaptation

The fitness dynamics across the concentration gradient of each constituent (ion) were evaluated by curve fitting of a cubic polynomial with the following equation (Eq. 4).


(4) $$\mu \,\left(x\right)=a\,x^{3}+b\,x^{2}+c\,x+d$$

Here, *x* and *μ(x)* represent the concentration gradient of each constituent and the growth rate under the corresponding conditions, respectively. *a*, *b*, *c* and *d* are the constants. The area under the regression curve was calculated according to the following equation (Eq. 5).


(5) $$A\,r\,e\,a=\int_{x_{m\,i\,n}}^{x_{m\,a\,x}}a\,x^{3}+b\,x^{2}+c\,x+d$$

Here, *x*_*min*_ and *x*_*max*_ represent the minimum and maximum concentrations of each chemical component, respectively. The space (***S***) was evaluated by normalizing both the height and the width of the regression curve with the following equation (Eq. 6).


(6) S=A⁢r⁢e⁢a×μm⁢a⁢x-1×(xm⁢a⁢x-xm⁢i⁢n)-1

Here, *μ_*max*_* is the maximal growth rate across the concentration gradient. The niche broadness (*S*_*T*_) of the individual genome was determined as the sum of the niche spaces of the eight chemical components as follows (Eq. 7).


(7) $$S_{T}=\sum_{i=1}^{n}S_{i}$$

Here, *S*_*i*_ and *n* indicate the niche space of each chemical component and the total number of chemical components, respectively.

### Genome Resequencing and Mutation Analysis

The stored cell culture was inoculated into 4 mL of fresh M63 medium in a test tube and grown at 37°C with shaking at 200 rpm. Once cell growth reached the stationary phase (OD_600_ > 1.0), rifampicin was added to the culture at a final concentration of 300 μg/mL to stop genome replication initiation. After 3 h of culture with rifampicin, the cells were collected as previously reported (Kishimoto et al., 2010). Genomic DNA was extracted using a Wizard Genomic DNA Purification Kit (Promega, United States) in accordance with the manufacturer’s instructions. The sequencing libraries were prepared using the Nextera XT DNA Sample Prep Kit (Illumina), and paired-end sequencing (300 bp × 2) was performed with the Illumina MiSeq platform. The sequencing reads were aligned to the *E. coli* W3110 reference genome (AP009048.1, GenBank), and the genome mutations were analyzed with the Breseq pipeline (version 0.30.1) (Barrick et al., 2014). The statistical data of DNA sequencing and mapping are summarized in Supplementary Table 5. The raw data set was deposited in the DDBJ Sequence Read Archive under accession number DRA011629. The fixed mutations (Supplementary Table 6) were subsequently analyzed for the temporal order of accumulation during evolution.

### Sanger Sequencing and Single-Colony Isolation

The genomic region of approximately 300–600 kb centered on the position of the mutation was amplified by PCR with PrimeSTAR HS DNA Polymerase (TaKaRa Bio, Japan) and the corresponding primers (Supplementary Table 7). Amplicons were purified using a MinElute PCR Purification Kit (Qiagen, United States), and Sanger sequencing was conducted by Eurofins Genomics K. K. (Tokyo, Japan). The resulting electropherogram was analyzed using Sequence Scanner Software v2.0 (Thermo Fisher Scientific, United States), and the ratio of the mutants within the cell population was calculated according to the peak values, as described previously (Kishimoto et al., 2015). Stored cell cultures with an interval of ∼100 generations were analyzed to identify the heterogeneity of the cell population. Single-colony isolation was performed from the heterogeneous population to isolate the homogeneous mutants. The cell culture was spread on LB agar plates, and 10∼30 single colonies per plate were subjected to Sanger sequencing. The colonies of the homogeneous mutant were stored for the fitness assay as described above.

## Data Availability Statement

The datasets presented in this study can be found in online repositories. The names of the repository/repositories and accession number(s) can be found in the article/Supplementary Material.

## Author Contributions

MK and IN performed the experiments. MK and B-WY analyzed the data and drafted the manuscript. B-WY conceived the research and rewrote the manuscript. All authors approved the final manuscript.

## Conflict of Interest

The authors declare that the research was conducted in the absence of any commercial or financial relationships that could be construed as a potential conflict of interest.

## Publisher’s Note

All claims expressed in this article are solely those of the authors and do not necessarily represent those of their affiliated organizations, or those of the publisher, the editors and the reviewers. Any product that may be evaluated in this article, or claim that may be made by its manufacturer, is not guaranteed or endorsed by the publisher.
