# Supplementary material for: Experimental Evolution Expands the Breadth of Adaptation to an Environmental Gradient Correlated With Genome Reduction
Source: Front Microbiol. 2022 Jan 26;13:826894. doi: 10.3389/fmicb.2022.826894 (PMC8826082; doi:10.3389/fmicb.2022.826894)
Supplement: Supplementary file 1 [file Presentation_1.PDF]

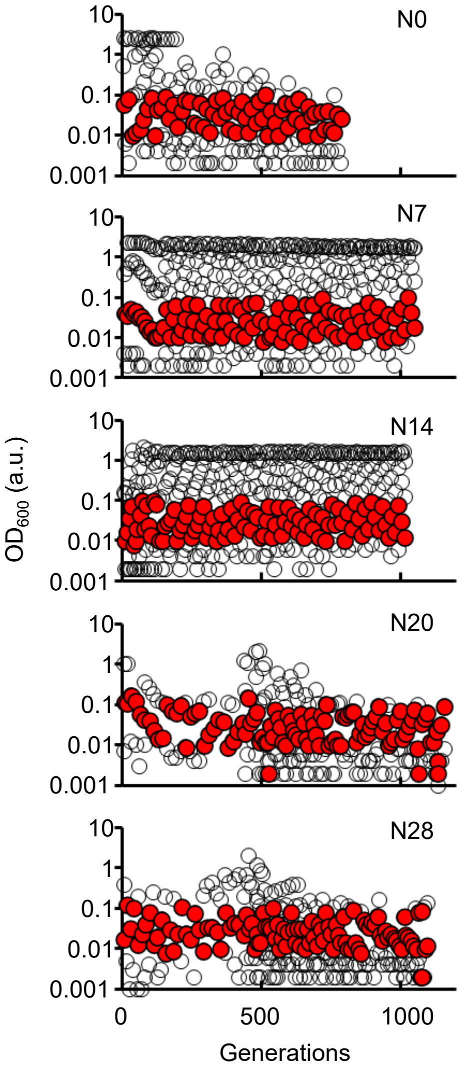

Figure S1 Temporal changes in OD_{600} during the experimental evolution. The grey and red circles indicate the eight cultures and the one selected for serial transfer, respectively. The OD_{600} of the selected cultures roughly ranged from 0.01 to 0.1. The genomes are indicated.

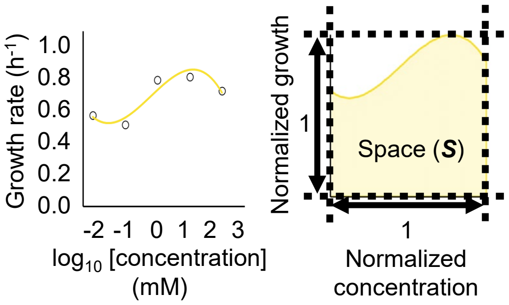

Figure S2 Definition of the space. The left and right panels indicate the regression curve of N7 (as an example) across the concentration gradient of glucose and the normalized regression curve, in which both the concentration gradient and the growth rates are rescaled within one unit, respectively. The area in shadow was determined as the space (***S***) of N7 in the niche of glucose.

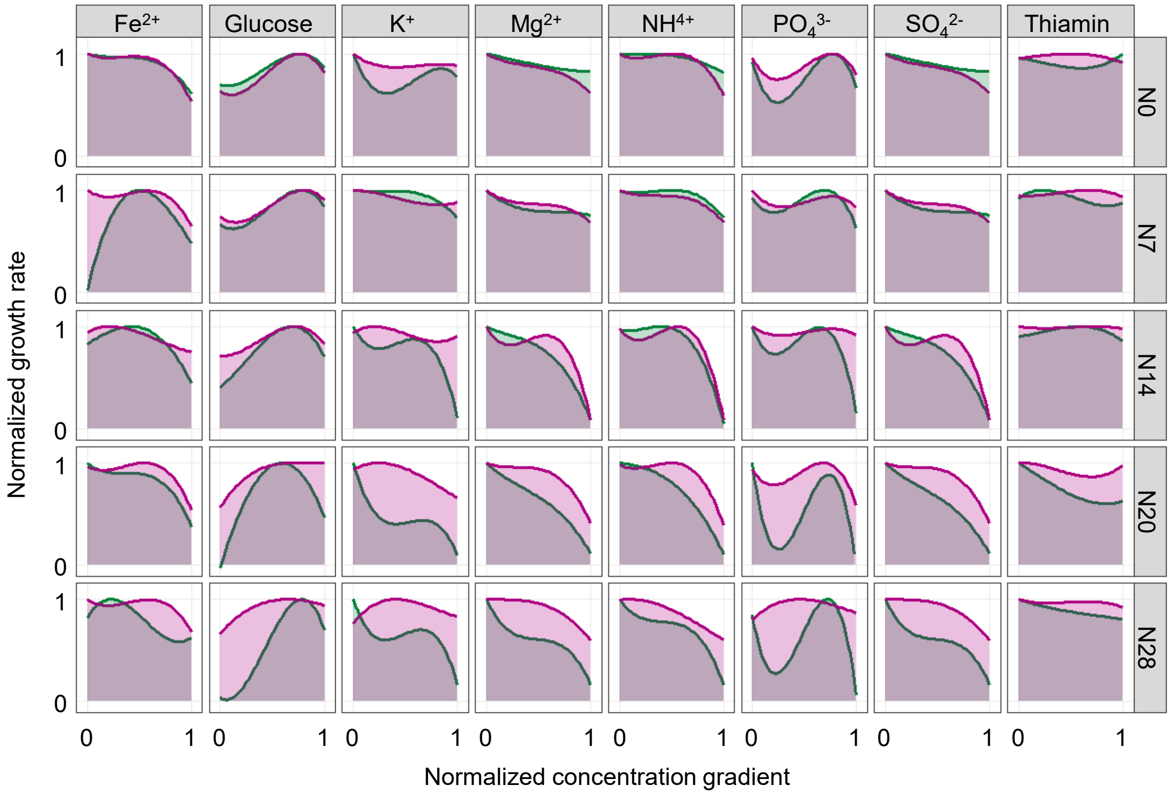

Figure S3 Fitted spaces of Ancs and Evos. The fitting curves are normalized, in which both the concentration gradient and the growth rates are rescaled within one unit. The transparent green and purple areas indicate the spaces (***S***) of Ancs and Evos, respectively.

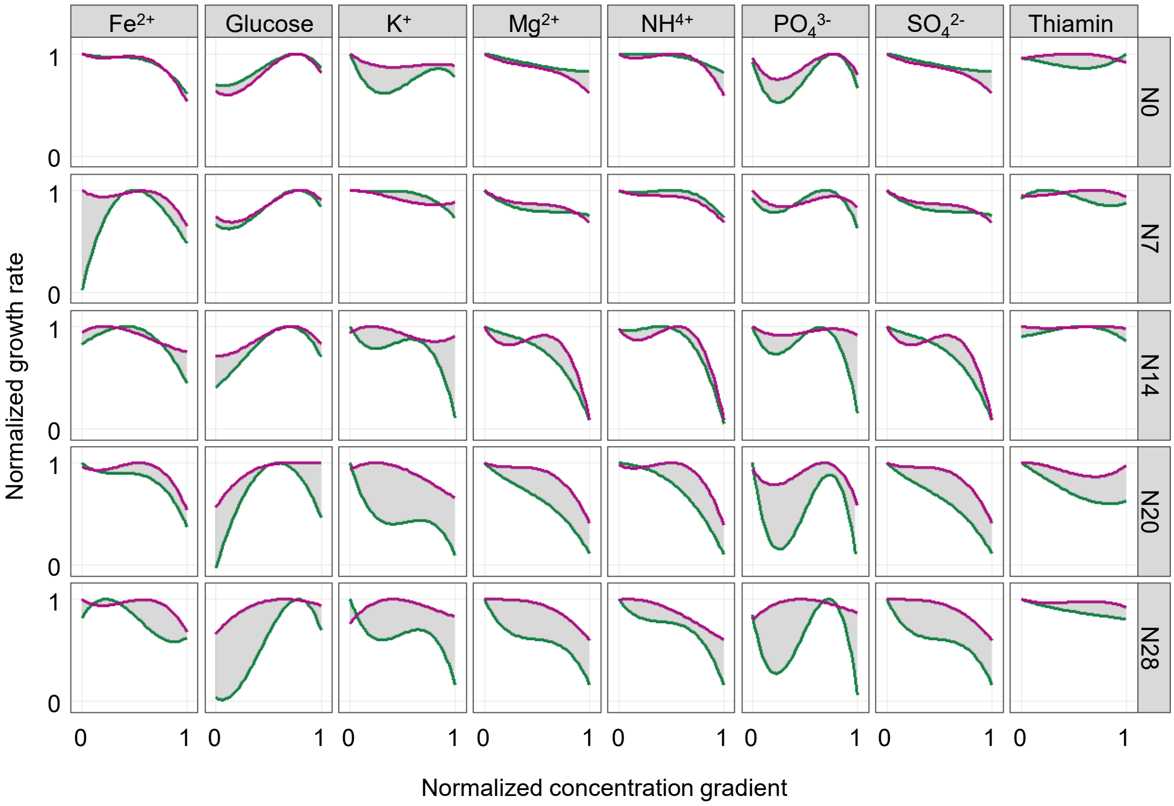

Figure S4 Changes in spaces between Ancs and Evos. The green and purple lines indicate the fitting curves of Ancs and Evos, respectively. The shadowed areas represent the changes in spaces (***S***) between Ancs and Evos.

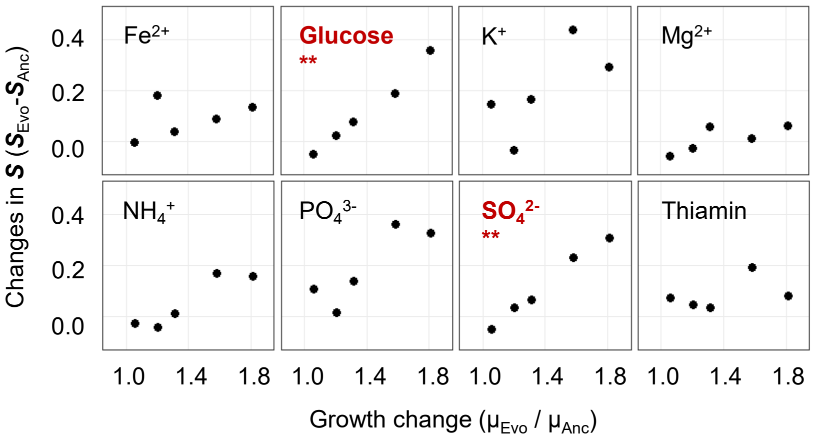

Figure S5 Relationships between the changes in *S* and the changes in local adaptation. The evolutionary changes in **S** of the five genomes are plotted against the changes in growth rate in C0 with respect to the eight chemical niches. The chemical niches and the statistical significance of the Spearman rank correlation are indicated. Boldfaces in red associated with asterisks represent statistical significance (*, $p < 0.05$; **, $p < 0.01$).

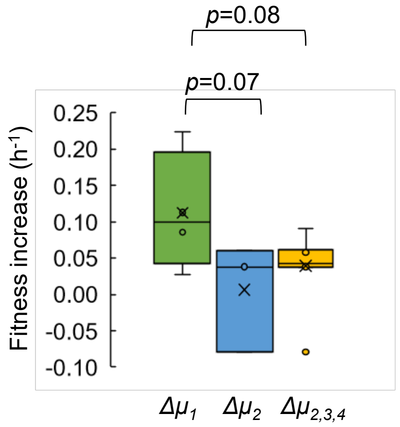

Figure S6 Boxplot of the changes in growth rates caused by mutation accumulation. Green, blue, and yellow represent the changes in growth rates caused by the first mutations, the second mutations, and the second, third and fourth mutations in the four genomes (N7, N14, N20 and N28), respectively. The statistical significance is indicated.

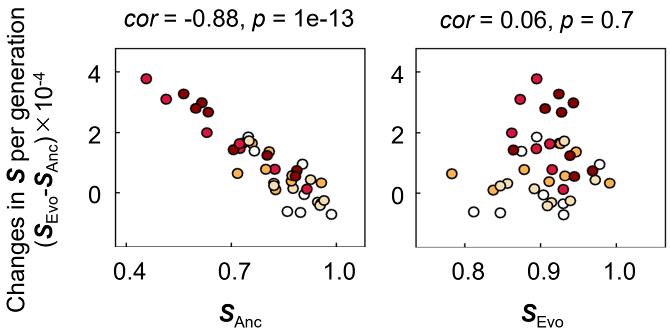

Figure S7 Relationships of $\boldsymbol{S_{Anc}}$ and $\boldsymbol{S_{Evo}}$ to the rate of changes in spaces. The rates of changes in the space (***S***) per generation are plotted against $\boldsymbol{S_{Anc}}$ and $\boldsymbol{S_{Evo}}$ in the left and right panels, respectively. The colour variation from white to dark red represents the five different genomes of N0~N28. The Spearman rank correlation coefficients and statistical significance are indicated.
